# The effect of CT and MRI with and without arthrography on the appearance of the feline carpal ligaments

**DOI:** 10.1186/s12917-022-03463-6

**Published:** 2022-10-07

**Authors:** Rachel M. Basa, Kenneth A. Johnson, Juan M. Podadera

**Affiliations:** Sydney School of Veterinary Science, Faculty of Science, University of Sydney, Camperdown, NSW 2006 Australia

**Keywords:** Magnetic resonance imaging, Feline, Carpus, Ligaments, Arthrography, Canine, Computed tomography

## Abstract

**Background:**

The current diagnosis of feline carpal injuries is based on radiographic examination including stress views and computed tomography; however, these techniques do not allow for direct evaluation of the carpal ligaments. The purpose of this cadaveric study was to assess the ability of CT arthrography (CTA) and MR arthrography (MRA) to provide this information using a single contrast mixture. A protocol for intra-articular injection of the feline carpus was also described. A contrast solution containing gadolinium and iohexol with a 50% gadolinium solution (Magnevist—gadolinium 0.5 mmol/mL diluted to a 0.05 mmol/mL solution) and 50% of iodine (Iohexol—iodine 300mgI/mL) was injected into the antebrachiocarpal and middle carpal joints of feline carpi using fluoroscopic guidance.

**Results:**

CTA allowed for identification of intra-articular ligaments and the silhouette of select extra-articular ligaments when there was adequate joint distension, however it was not considered to be superior to MRI. MRA allowed for improved identification of the dorsal radiocarpal, accessorioulnocarpal, accessorioquartile, short ulnar and short radial collateral ligaments.

**Conclusion:**

In this ex-vivo study, combined CTA and MRA enhanced the appearance of the feline carpal ligaments and may provide a foundation for future studies in the diagnosis of carpal injuries.

**Supplementary Information:**

The online version contains supplementary material available at 10.1186/s12917-022-03463-6.

## Background

Feline carpal injuries are often caused by falling from a height or motor vehicle accidents. Whilst in the dog the carpometacarpal joint is most commonly injured, in the cat there is a higher prevalence of these injuries affecting the antebrachiocarpal, followed by the carpometacarpal and middle carpal joints [1–4]. Antebrachiocarpal subluxation and luxation in the cat is possible with rupture of the short radial collateral ligament and dorsomedial joint capsule alone [2, 5]. In the absence of hyperextension injury, or the rare case where there is isolated ligament injury or carpal bone fracture, arthrodesis may be avoided in order to maintain normal carpal joint range of motion. Arthrodesis is considered to be a salvage procedure that aims to achieve osseous fusion of the carpal joint spaces, minimizing the chance of pain originating from an unstable joint [6, 7].

Given the complexity of the anatomy and the small size of the joint components, the study of the feline carpus represents a major diagnostic challenge. The current standard of care in the diagnosis of feline carpal joint injuries is based on radiographic examination and computed tomography. Radiographic examination includes standard orthogonal projections (dorsopalmar and mediolateral projections) and views with medial, lateral, dorsal and palmar stress in order to assess the stability of the joint and the integrity of the collateral ligaments, palmar ligaments and joint capsule. The disadvantage of these techniques is that they do not allow for direct identification of the carpal ligaments, which may limit the ability of the treating clinician to offer primary ligament repair instead of arthrodesis. The decision to perform either partial or pancarpal arthrodesis depends on the level of joint instability and ligaments involved [6, 7].

High field MRI has previously been used to describe the appearance of the feline carpal ligaments [8]. In a clinical setting, the availability of high field MRI may be limited to certain referral institutions and computed tomography (CT) is considered to be a faster, less expensive and more globally available imaging modality. The utility of CT in describing pathology of complex joints such as the carpus can be improved by using arthrography. The use of contrast arthrography is reported in the canine carpus, shoulder and stifle, but to the knowledge of the authors there are no similar studies in the feline carpus [9–12].

The aims of this study were therefore to describe a protocol for intra-articular injection of the feline carpus, and to assess the ability of CTA and MRA compared with plain CT and MRI to allow for direct evaluation of the carpal ligaments using a single contrast mixture. In addition, this manuscript documents normal joint communications during fluoroscopic injection of the feline carpus.

## Results

### Pilot study

#### Volume and concentration of contrast

There was sufficient distension with 0.8-millilitre volume within the antebrachiocarpal joint and 0.5 ml within the middle carpal joint. Solution 3 was assessed to have the most appropriate concentration of contrast, with solution 1 showing evidence of beam hardening artefact. Solution 2 had a lower volume of gadolinium resulting in less conspicuous enhancement of the synovial fluid. The mean signal intensity was recorded by assessing the number of Hounsfield units on CT, and density on MRI in three different locations within the scan, as measured using medical imaging software (OsiriX, Pixmeo SARL, Bernex, Switzerland) (Additional file 1 Appendix Table 1). Solution 3 had the lowest number of Hounsfield units, and solution 2 had the lowest recorded intensity.

#### Distribution of contrast with fluoroscopy

**Fig. 1:**
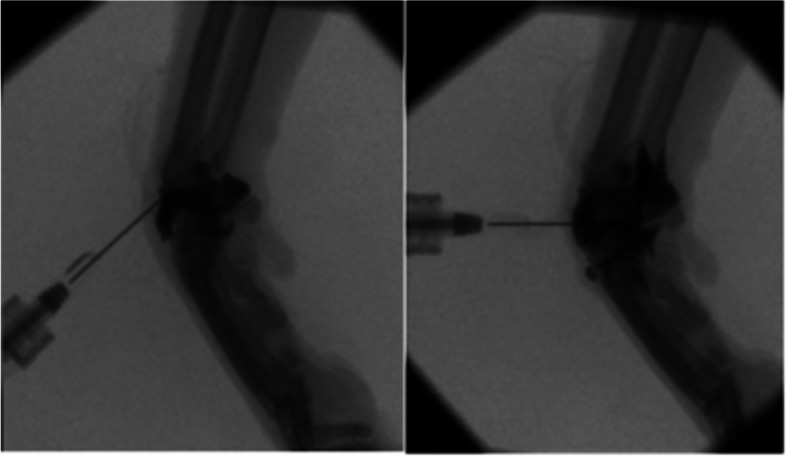
Still fluoroscopic images shown from the feline carpus in the sagittal plane. The image on the left shows contrast distribution while injecting the antebrachiocarpal joint. The image on the right shows contrast distribution while injecting the middle carpal joint, after injecting the antebrachiocarpal joint

Contrast filling of the antebrachiocarpal joint tended to occur dorsally and distally first, with the palmar joint pouch filling last with contrast material (Fig. 1). It was difficult to determine the distribution of contrast material within the middle carpal joint due to the tendency for contrast to extend distally from the antebrachiocarpal joint. There was consistently no communication between the antebrachiocarpal and middle carpal joints within all of the specimens.

#### Ligament visibility: computed tomography compared with computed tomography with arthrography

**Fig. 2:**
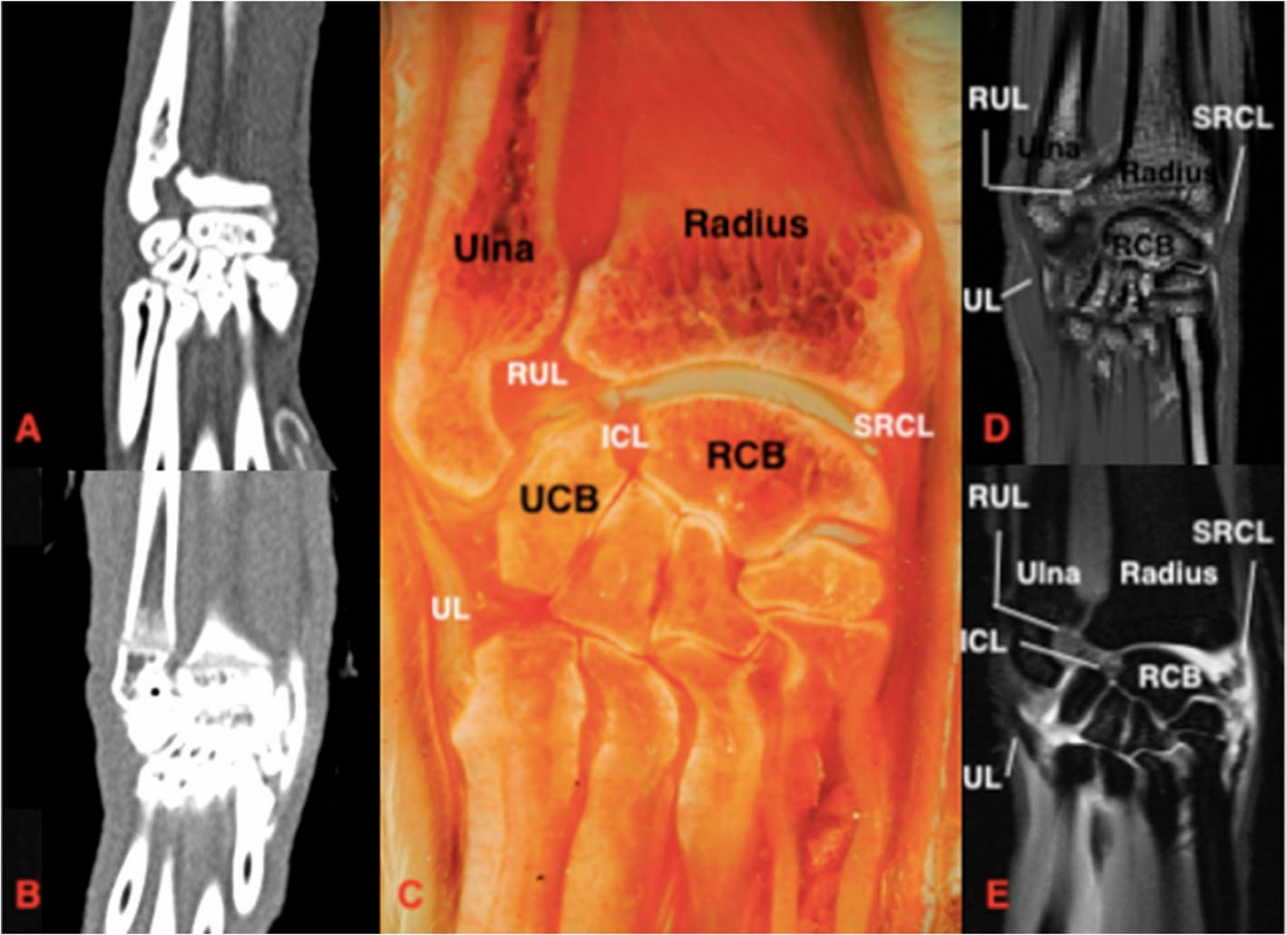
Dorsal section of the feline carpus. Proximal is at the top of the image and lateral is to the left.** A** Plain CT, soft tissue window **B**) CTA, soft tissue window **C**) Gross plastinated section **D**) T1W **E**) T1W FS post contrast. Note that the silhouette of the SRCL can be seen in **B**) post contrast injection. There is improved visibility of the SRCL in the post contrast MRI (**E** compared to **D**). RCB: radial carpal bone, UCB: ulnar carpal bone, RUL: radioulnar ligament, UL: ulnaris lateralis, ICL: radioulnar intercarpal ligament, SRCL: short radial collateral ligament

**Fig. 3:**
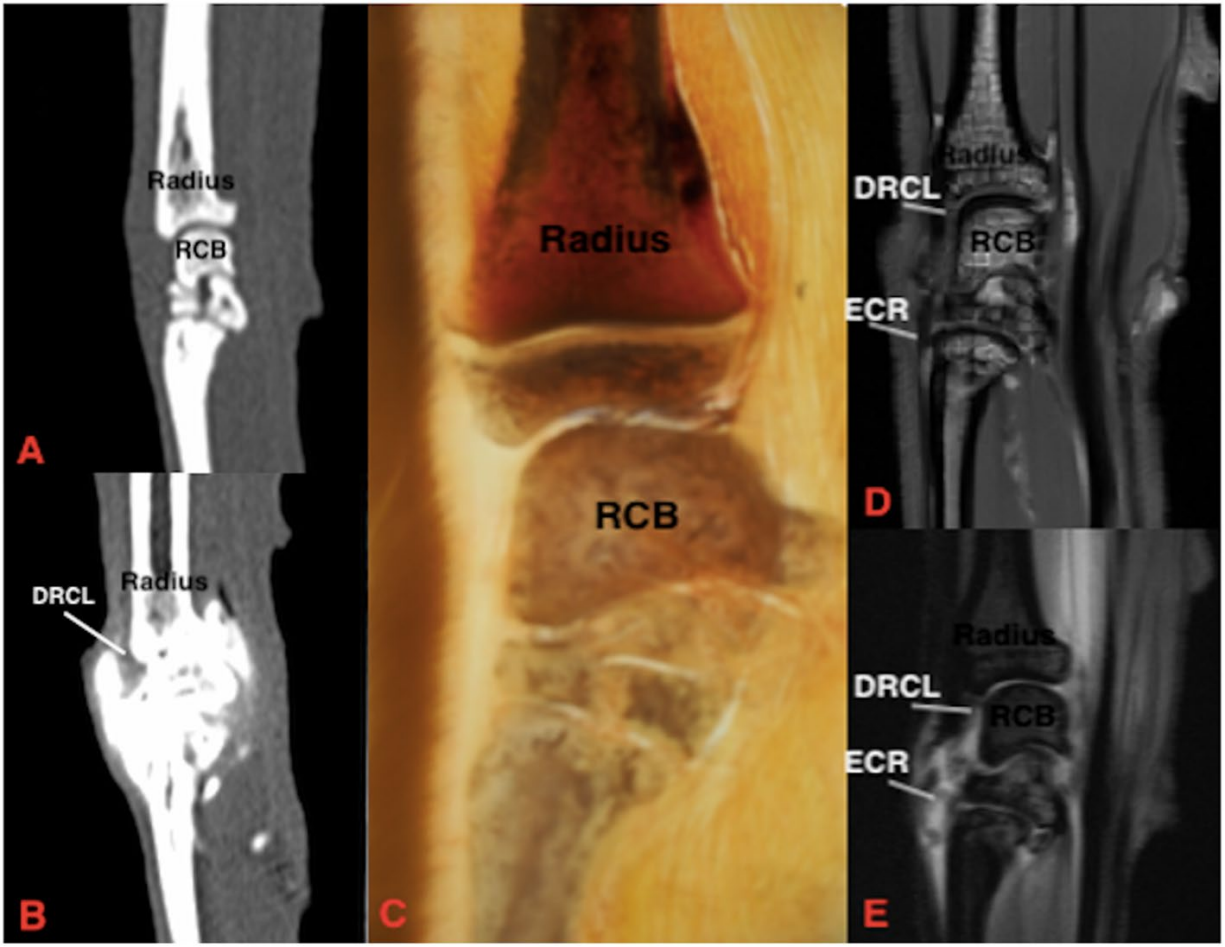
Sagittal section of the feline carpus. Dorsal is to the left of the image and proximal is at the top. **A** Plain CT, soft tissue window **B**) CTA, soft tissue window **C**) Gross plastinated section **D**) T1W **E**) T1W FS post contrast. Note that no ligaments are visible with plain CT, and the silhouettes of select ligaments and tendon are seen with distension of the joint capsule using CTA. DRCL: dorsal radiocarpal ligament, ECR: extensor carpi radialis tendon

**Fig. 4:**
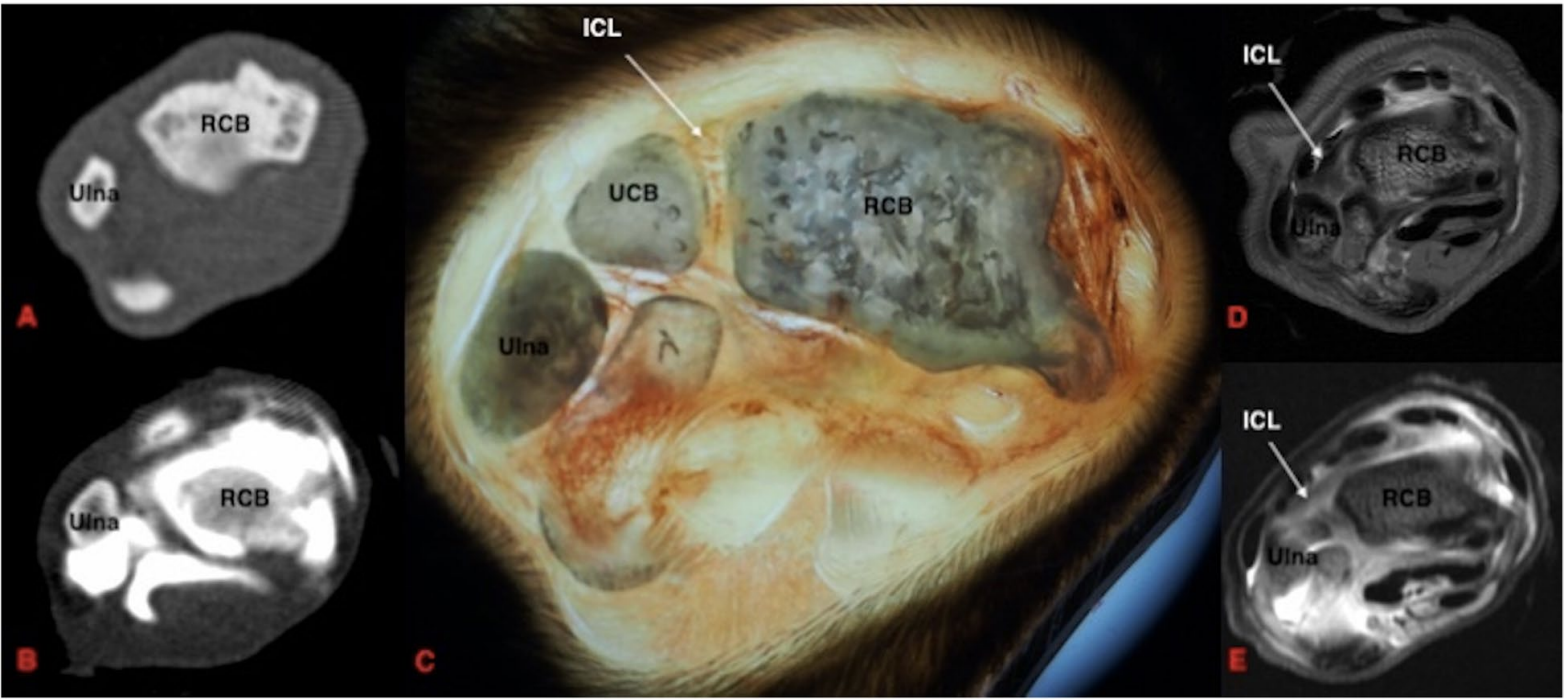
Transverse section of the feline carpus, distal to the antebrachiocarpal joint. Dorsal is at the top of the image and lateral is to the left. **A** Plain CT, soft tissue window **B**) CTA, soft tissue window **C**) Gross plastinated section **D**) T1W **E**) T1W FS post contrast. In **B** and **E** it can be noted that there is contrast extravasation due to over-distension of the antebrachiocarpal joint. RCB: radial carpal bone, UCB: ulnar carpal bone, ICL: radioulnar intercarpal ligament

None of the ligaments could be identified using plain CT. With the use of CTA, only the contours of ligaments that were surrounded by contrast enhanced synovial fluid were readily identified based on multi-planar reconstruction, including the inter-carpal ligaments, short radial collateral ligament, palmar ulnocarpal ligament and accessoriometacarpal ligament (Figs. 2, 3, and 4) and short ulnar collateral ligament. The palmar ulnocarpal ligament was easily identified, especially in the dorsal reconstruction. The palmar radiocarpal ligament, accessorioulnocarpal ligament and accessorioquartile ligaments could not be seen. Adequate distension of the dorsal joint pouch enabled identification of the extra-articular short radial collateral (Fig. 2) and short ulnar collateral ligaments.

#### Ligament visibility: magnetic resonance imaging compared to magnetic resonance imaging with arthrography

**Fig. 5:**
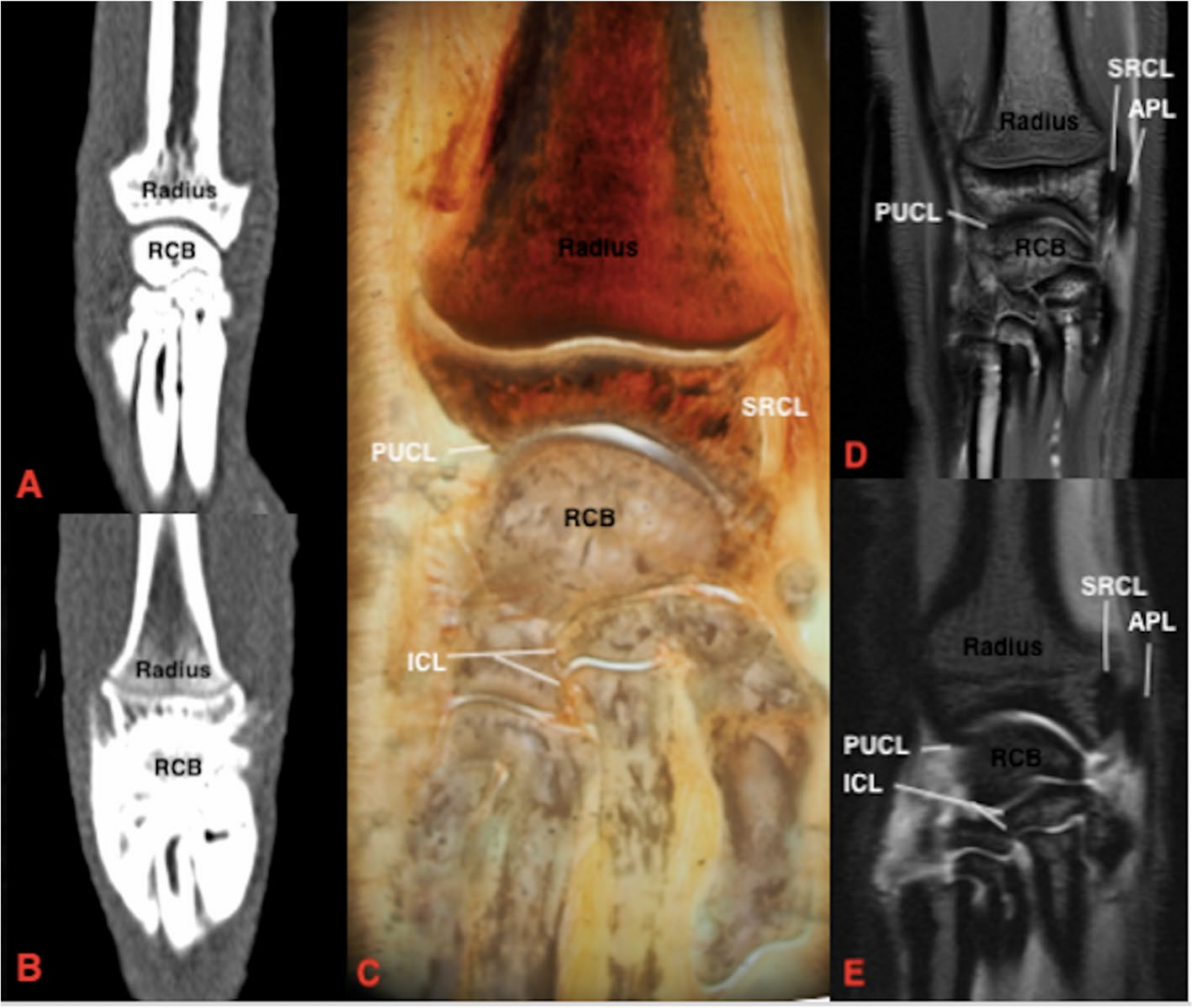
Dorsal section of the feline carpus. Proximal is to the top of the image and lateral is to the left. **A** Plain CT, soft tissue window **B**) CTA, soft tissue window **C**) Gross plastinated section **D**) T1W **E**) T1W FS post contrast. In **B** there is no extension of contrast proximal to the radial articular surface due to the presence of the SRCL, APL and ICL. RCB: radial carpal bone, ICL: intercarpal ligament, PUCL: palmar ulnocarpal ligament, SRCL: short radial collateral ligament, APL: abductor pollicus longus tendon

Both intra and extra-articular ligaments were identified using MRI (Figs. 2, 3, 4, and 5). Using MRA the ligaments were seen as hypo-intense linear bands surrounded by hyper-intense (contrast enhancing) synovial fluid. The T1 and T1 sequences with fat suppression subjectively had a similar appearance. A summary of ligament visibility in different imaging planes in provided in Table 1. The ligaments that were visible in all imaging planes included the palmar radiocarpal and radioulnar ligaments.

**Table 1:** Means of ligament visibility scores between five feline carpi, pre and post MRA

	Sagittal		Dorsal		Transverse	
	MRI Pre contrast	MRA Post contrast	MRI Pre contrast	MRA Post contrast	MRI Pre contrast	MRA Post contrast
RUL	0.3	0.3	3	3	3	3
PRML	3	3	0	0	2.7	2.7
DRCL*	2.3	2.7	1.7	2.3	0	0
ICL	0	0	3	3	3	3
SUCL*	0	0	2.7	2.7	0	0.3
SRCL*	0	0	2.7	3	2.3	3
PUCL*	1	1.7	1.7	2.7	0	0.3
PRCL*	2.3	3	2	2.3	3	3
AUL*	1	1.7	0	0	2.7	3
AML	3	3	0	0	2.7	2.7
AQL*	1	2	3	3	0	0

*The ligaments with which contrast improved the visibility score are marked with an asterix

*Notes*: *RUL* radioulnar ligament, *PRML* palmar radiocarpal metacarpal ligament, *DRCL* dorsal radiocarpal ligament, *ICL* radioulnar intercarpal ligament, *SUCL* short ulnar collateral ligament, *SRCL* short radial collateral ligament, *PUCL* palmar ulnocarpal ligament, *PRCL* palmar radiocarpal ligament, *AUL* accessorioulnocarpal ligament, *AML *accessoriometacarpal ligament, *AQL* accessorioquartile ligament

The intra-articular ligaments include the radioulnar, intercarpal, palmar radiocarpal and palmar ulnocarpal ligaments. The radioulnar and intercarpal ligaments were clearly visible in both the dorsal and transverse planes prior to contrast injection. These ligaments are short thick bands with a transverse orientation and were unable to be identified clearly or at all in the sagittal plane. The palmar radiocarpal ligament was easiest to view in the transverse plane. MRA improved the visibility of the palmar radiocarpal and palmar ulnocarpal ligaments in both sagittal and dorsal planes.

The extra-articular ligaments include the palmar radiocarpal metacarpal ligament, dorsal radiocarpal ligament, short ulnar collateral ligament, short radial collateral ligament and ligaments of the accessory carpal bone. MRA improved the visibility of the accessorioquartile ligament in the sagittal plane but made no difference to visibility in the remaining planes. The visibility of the accessorioulnocarpal ligament was improved in the sagittal and transverse planes. The short ulnar collateral ligament and short radial collateral ligaments were unable to be seen in the sagittal plane. MRA enhanced visibility of the short radial collateral ligament in the transverse plane. The palmar radiocarpal and accessoriometacarpal ligaments were both readily identified with both MRI and MRA in the sagittal and transverse planes. MRA improved the visibility of the dorsal radiocarpal ligament in both sagittal and dorsal planes.

## Discussion

Based on the results of this study, CTA and MRA improved the identification of intra-articular ligaments of the feline carpus. The silhouette of extra-articular ligaments were not consistently identified with CTA. MRA improved visibility of the palmar ulnocarpal, palmar radiocarpal and accessorioquartile ligaments. Select extra-articular ligaments also had improved visibility with the use of MRA, particularly the dorsal radiocarpal ligament which is closely associated with the dorsal carpal joint capsule.

Arthrography can be performed blindly or using fluoroscopy, sonography, CT or MRI guidance [13–16]. Most previously reported arthrogram studies in the dog have used a blind injection technique, whereby the needle is inserted within the joint space based on known anatomical landmarks [9, 10]. In the feline carpus where there are smaller joint spaces, accuracy in needle placement may be more challenging. Fluoroscopy was used to confirm needle placement in the current study and allowed for the visualization of contrast distribution during injection in real time [15].

Due to there being no communication between the antebrachiocarpal and middle carpal joints, these were separately injected with contrast. There was a tendency for contrast to travel dorsodistally in the feline antebrachiocarpal joint, therefore there was contrast material that overlapped the dorsal aspect of the middle carpal joint. Based on this observation, it is advisable to inject the middle carpal joint prior to the antebrachiocarpal joint in the cat in order to avoid contrast leakage as a result of puncture of the dorso-distal pouch of the antebrachiocarpal joint. The authors acknowledge that the direction and depth of the bevel of the needle may also play a role in the distribution of contrast. Distally, it was noted that there was contrast within the inter-carpal joint between the third and fourth carpal bones, but not the second and third carpal bones. This is consistent with previous reports in the dog, where there is no inter-carpal ligament between the third and fourth carpal bones [17].

A pilot study was performed to determine the optimal volume and concentration of contrast solution. In the human wrist, the general recommendation is to inject 2–3 millilitres of contrast within the radiocarpal joint, and 1 ml within the distal radioulnar joint [15]. There are no such recommendations in small animals, therefore the authors recommend injecting each joint until there is soft palpable capsular distension. A potential disadvantage of relying on the presence of capsular distension is that this is associated with pain in people and may be difficult to quantitate in small animal patients [16].

Previous human studies have assessed the optimal concentration of a gadolinium/ iodinated mixed contrast solution [18, 19]. High concentration of iodinated contrast material on MRA compromises the signal intensity derived from gadolinium, resulting in decreased signal intensity on T1 weighted images [18, 19]. Lower concentrations of iodine may result in lower solution viscosity that may enhance the delineations of small intra-articular structural irregularities. Based on the results of the pilot study, the optimal contrast solution (see Additional file 1 Appendix Table 2, solution 3) contained 1.25 mmol/L gadolinium concentration (1/400 part of the Magnevist 0.5 mol/L) and 150 mg/ml of iodine.

Ligaments were identified using CTA by assessing the contours that were highlighted due to the presence of surrounding contrast agent. CTA was considered more valuable at assessing distribution of contrast, demarcating joint margins and joint communications, which may be abnormal in the presence of inter-carpal ligament or capsular tears. Compared with MRA, CTA has the benefit of being readily available and faster to perform. It also provides better evaluation of thin cortical bone and subtle calcifications; however, the level of soft tissue contrast is inferior to MRI [13, 20].

CTA has also been studied in the normal canine carpus, and it was noted to be superior at identifying the radioulnar ligament and palmar radiocarpal ligament compared to CT alone [12]. In the current study, it was possible to identify the silhouette of the radioulnar intercarpal ligament, short radial collateral ligament, short ulnar collateral ligament, palmar ulnocarpal and accessoriometacarpal ligaments using CTA, none of which were visible with plain CT. Further studies would be required to identify the benefits of using CTA in the injured carpal joint. This technique has been used effectively in wrist injuries to determine the location of intra-articular ligament tear and perforation [21].

The T1 weighted sequences are used in MRA because they allow for the visibility of contrast. The addition of the T1 fat suppression sequence reduces adipose tissue signal, therefore resulting in a relative increase in contrast conspicuity [9]. The reported benefits of MRA compared to conventional MRI include that all joint cavities are fully expanded, which means that the joint capsule is distended so that neighbouring ligaments can be more conspicuous. The high signal of gadolinium leads to improved delineation of ligaments, which inherently have low signal intensity [10, 22]. In the human wrist, arthrography has been confirmed to increase the sensitivity of identifying inter-carpal ligament tears and various ligaments of the wrist [22, 23]. In the present study, MRA improved the visibility of the palmar radiocarpal, palmar ulnocarpal, accessorioulnocarpal, accesorioquartile, short radial collateral, short ulnar collateral and dorsal radiocarpal ligaments in most imaging planes.

The limitations of this study include that cadaveric limbs that underwent a freeze thaw cycle were the test subjects, which may have resulted in hydrogen atom loss in the form of water, and potential for imaging artifact [24]. Furthermore, ligament visibility was assessed by two observers who were not blinded to the study design and there may be interobserver variability that was not accounted for. The study would also require a greater number of cases to be able to carry out statistical evaluation of the data and determine whether there is a significant different in ligament visibility.

## Conclusion

CTA may be particularly useful to the treating clinician where there is restricted access to high field MRI. CTA should not replace manual palpation and stress radiographs to assess for carpal joint instability, however it may be helpful in cases with concurrent carpal bone fracture, or where there is uncertainty regarding the integrity of the joint capsule and collateral or intra-articular ligaments. MRA may be advantageous compared to MRI where there is concern regarding the palmar stabilisers of the carpus. It can be particularly difficult to diagnose hyperextension injury based on orthopaedic examination immediately post injury because a palmigrade stance may be masked by non- weight bearing lameness.

In conclusion, CTA provides more information than CT alone however MRI and MRA both provide superior contrast resolution when assessing the feline carpal ligaments. Further research and application of this study methodology with multiple blinded observers would be required before it is routinely used in clinical cases of feline carpal injury.

## Methods

### Subject selection

Five paired feline cadaveric forelimbs were harvested from mature cats after they had been euthanised for reasons unrelated to the study in accordance with guideline GL001 from the University of Sydney animal ethics committee. The cadavers had previously been used for teaching purposes through the Sydney School of Veterinary Science, and no further permissions were required. Sample size was based on methodology from previous studies [8, 25]. The cats were Domestic Short Hair ranging in size from 3.5–5.0 kg. The individual limbs were selected for study inclusion based on the results of a random number generator. Inclusion criteria included that the animals were skeletally mature. Exclusion criteria included the presence of suspected soft tissue abnormality on MRI.

### Pilot study

In brief, a pilot study was conducted in order to determine appropriate contrast concentration for combined CTA and MRA. Three canine antebrachii were used to assess the concentration of contrast agent, with varying quantities of Omnipaque (iohexol 350mgI/mL) and Magnevist (gadopentetate meglumine 0.469 g/mL, 0.5 mol/ml) (Additional file 1 Appendix Table 2); these concentrations were based on the appearance of phantoms used in a previous human study [18]. MRA was conducted using the technical parameters described in Table 2. The CT parameters were mAs 50, KVp 90, slice thickness 0.75 mm, matrix 512*512, pitch of 0.5 using an ultra-high-resolution filter and bone algorithms; the same parameters were used in the final study.

**Table 2:** MRI specifications used to image the feline carpus

	Study MRI
Sequence	T1, T1 fat suppression
Flip angle (degrees)	111
Repetition time (ms)	713
Echo time (ms)	12.3
Field of view (mm)	50
Matrix (number of pixels)	320 × 320
Slice thickness (mm)	1.3
Inter-slice gap	0.2
Number of excitations	12

### Preparation of study limbs

Five feline antebrachii were selected for study inclusion. The carpi were positioned with 180 degrees of extension on a perspex sheet between imaging modalities.

### Plain computed tomography and magnetic resonance imaging

Plain CT and MRI were initially performed on each of the feline carpi (*n*=5).

Using CT (16 slice Philips Brilliance helical CT, Amsterdam, Netherlands) the limbs were scanned with the following parameters: 120KV, 200mAs, slice thickness 0.8mm and ultra- high resolution.

A 3 Tesla MRI (GE Healthcare Milwaukee, USA, Model Discovery MR750) was also used and the limbs were positioned with the carpus in extension, using a human volume coil. T1 and T1 with fat suppression pre and post intra-articular contrast administration were performed.

### Fluoroscopy

Prior to fluoroscopy, 5 millilitre syringes were attached to an extension set and needle. Each was primed with a pre-mixed contrast solution. The carpus was flexed and a 25- gauge needle was first inserted into the antebrachiocarpal joint, with needle placement being confirmed using fluoroscopy in both frontal and sagittal projections. The needle was considered to be within the antebrachiocarpal joint space when in the frontal plane, the bevel of the needle was proximal to the central aspect of the articular surface of the radial carpal bone and below the radial articular surface, within the joint space. Once needle placement was confirmed, 1–2 ml of contrast was injected into the feline antebrachiocarpal joints and 0.5–1 ml of contrast in the feline middle carpal joints. The contrast was firstly injected within the antebrachiocarpal joint until there was dorsal and proximal extension of contrast material within the synovial pouches. The needle was within the middle carpal joint space when it was confirmed to be distal to the radial carpal bone and proximal to carpal bones III and IV. Contrast material was then injected within the middle carpal joint until it was seen throughout the carpometacarpal joint spaces. Each carpus was gently flexed and extended to facilitate even distribution of contrast agent.

### Computed tomography and magnetic resonance imaging with arthrography

Following arthrography, CTA and MRA were performed with each limb maintained in the same position. All imaging planes (transverse, dorsal and sagittal) were acquired. In order to standardize positioning of the limbs between scans, anatomical landmarks were used to plan orientation lines of the limbs in each sequence.

### Image analysis

A board-certified veterinary radiologist (JMP) and board-certified veterinary surgeon (RMB) viewed all of the computed tomography (CT) and magnetic resonance images (MRI) using medical imaging software (OsiriX, Pixmeo SARL, Bernex, Switzerland) on a desktop (Mac Pro 3.5 GHz 6-core Intel Xeon), viewed with a medical monitor Eizo RX 340. The CT scans were reconstructed into multiple planes using the same anatomical landmarks as the MRI sequences.

The ligaments were identified in each scan using previously reported feline carpal ligament anatomy as a guideline [8, 17]. The ligaments were also compared to gelatin embedded frozen specimens sectioned in transverse, dorsal and sagittal planes in addition to epoxy plastinated specimens that were used in a previous study [8]. The visibility of the carpal ligaments based on pre and post contrast MRI was characterized based on a visual assessment score that has been validated in a previous study [26]. The scoring system is as follows; 0: the ligament was not visible, 1: the ligament was partially identified, 2: the ligament was identified in its totality but poorly demarcated, 3: the ligament was totally identified and well demarcated [26].

## Supplementary Information

**Additional file 1.**

## Data Availability

The datasets used and/or analysed during the current study are available from the corresponding author on reasonable request.

## Abbreviations

AQL: Accessorioquartile ligament
AML: Accessoriometacarpal ligament
AUL: Accessorioulnocarpal ligament
PRCL: Palmar radiocarpal ligament
PUCL: Palmar ulnocarpal ligament
SUCL: Short ulnar collateral ligament
SRCL: Short radial collateral ligament
RUL: Radioulnar ligament
ECR: Extensor carpi radialis tendon
ICL: Radioulnar intercarpal ligament
PRML: Palmar radiocarpal metacarpal ligament
DRCL: Dorsal radiocarpal ligament
UL: Ulnaris lateralis
APL: Abductor pollicus longus tendon
RCB: Radial carpal bone
UCB: Ulnar carpal bone
CTA: Computed tomography arthrography
MRA: Magnetic resonance arthrography
FS: Fat suppression
STIR: Short tau inversion recovery
