# Supplementary material for: The effect of CT and MRI with and without arthrography on the appearance of the feline carpal ligaments
Source: BMC Vet Res. 2022 Oct 7;18:368. doi: 10.1186/s12917-022-03463-6 (PMC9540734; doi:10.1186/s12917-022-03463-6)
Supplement: Supplementary file 1 — Additional file 1. [file 12917_2022_3463_MOESM1_ESM.docx]

Appendix table 1

| *MRI pilot* | Gadolinium solution | Iohexol |
| --- | --- | --- |
| *Specimen 1* | 25% gadolinium (2.5mmol/L) =  0.03mls Magnevist (0.5mmol/ml) + 4.97mls saline | 75% iohexol  = 15mls 350mg I/ml |
| *Specimen 2* | 25% gadolinium (2.5mmol/L) =  0.03mls Magnevist (0.5mmol/ml) + 4.97mls saline | 75% iohexol (350mg I/ml)  12.75mls I + 2.25mls saline (300mg I/ml solution) |
| *Specimen 3* | 50% gadolinium (2.5mmol/L) =  0.06mls Magnevist (0.5mmol/mL) + 9.94mls saline | 50% iohexol  12.75mls I + 2.25mls saline (300mg I/ml solution) |

Appendix table 2

|  | Specimen 1 | Specimen 2 | Specimen 3 |
| --- | --- | --- | --- |
| Canine CTA  (Hounsefield units) | 2976 | 2976 | 2800 |
| Canine MRA  (Intensity) | 1434 | 1366 | 2529 |
